# Adolescent Oxytocin Exposure Causes Persistent Reductions in Anxiety and Alcohol Consumption and Enhances Sociability in Rats

**DOI:** 10.1371/journal.pone.0027237

**Published:** 2011-11-16

**Authors:** Michael T. Bowen, Dean S. Carson, Adena Spiro, Jonathon C. Arnold, Iain S. McGregor

**Affiliations:** 1 School of Psychology, University of Sydney, Sydney, Australia; 2 Brain and Mind Research Institute, University of Sydney, Sydney, Australia; 3 Department of Pharmacology, University of Sydney, Sydney, Australia; Sapienza University of Rome, Italy

## Abstract

Previous studies have suggested that administration of oxytocin (OT) can have modulatory effects on social and anxiety-like behavior in mammals that may endure beyond the time of acute OT administration. The current study examined whether repeated administration of OT to male Wistar rats (n = 48) during a key developmental epoch (early adolescence) altered their physiology and behavior in later-life. Group housed rats were given intraperitoneal injections of either 1 mg/kg OT or vehicle during early adolescence (post natal-days [PND] 33–42). OT treatment caused a transient inhibition of body weight gain that recovered quickly after the cessation of treatment. At PND 50, the rats pre-treated with OT displayed less anxiety-like behavior on the emergence test, while at PND 55 they showed greater levels of social interaction. A subgroup of OT pre-treated rats examined at PND 63 showed a strong trend towards increased plasma OT levels, and also displayed significantly increased OT receptor mRNA in the hypothalamus. Rats pre-treated with OT and their controls showed similar induction of beer intake in daily 70 min test sessions (PND 63 onwards) in which the alcohol concentration of beer was gradually increased across days from 0.44% to 4.44%. However, when given *ad libitum* access to beer in their home cages from PND 72 onwards (early adulthood), consumption of beer but not water was significantly less in the OT pre-treated rats. A “booster” shot of OT (1 mg/kg) given after 25 days of *ad libitum* access to beer had a strong acute inhibitory effect on beer intake without affecting water intake. Overall these results suggest that exogenous OT administered during adolescence can have subtle yet enduring effects on anxiety, sociability and the motivation to consume alcohol. Such effects may reflect the inherent neuroplasticity of brain OT systems and a feed-forward effect whereby exogenous OT upregulates endogenous OT systems.

## Introduction

The neuropeptide oxytocin (OT) regulates a number of critical behavioral repertoires in mammals including maternal behaviour [1]–[3], social interaction and social preference [4], [5], sexual behaviour [6], and anxiety-like behaviour [2]. The anxiolytic properties of OT, and related OT receptor agonists, have been documented in many different rodent models of anxiety and with many different routes of administration [7]–[11]. OT can also reverse some of the anxiogenic effects of social separation. For example, social interaction deficits in prenatally stressed rat pups were reversed by administering OT into the central amygdala [12]. Central administration of OT also reduced distress ultrasonic vocalisation production in rat pups during social isolation, perhaps by mimicking the effects of social contact [13]. In a more recent study, socially isolated voles given OT peripherally showed fewer lasting adverse effects of long-term social isolation than controls [14]. Furthermore, chronic central OT administration in male rats increased the duration and frequency of non-sexual physical contact with female rats, irrespective of their oestrous cycle, which suggests chronic OT may facilitate nonsexual social interactions [15]. The anxiolytic and prosocial effects of OT evident in these animal models have led to worldwide interest in exogenous OT as a potential therapeutic for human psychiatric disorders [2], [16]–[23].

In some published studies these effects of OT are more apparent with repeated, rather than acute, administration. Thus chronic, but not acute, centrally administered OT attenuated the pathological high anxiety of female rats selectively bred for high anxiety-related behaviour [24] while 3 days of peripheral OT treatment significantly reduced anxiety in rats following the induction of colitis [25]. In other studies, repeated peripheral OT treatment had lasting beneficial effects on blood pressure and pain tolerance [26], [27] and causes long-lasting decreases in blood concentrations of corticosterone [10], [28]. Even more striking, rats administered 1 mg/kg OT peripherally once per day from postnatal days 1–14 showed significantly reduced blood pressure in adulthood (aged 7–8 months) [29].

The ability of exogenous OT to cause enduring residual changes in behavioral and physiological traits may reflect the inherent plasticity of OT neural systems. Heightened stimulation of hypothalamic OT receptors (OTRs) triggers increased dendritic and peripheral release of OT which further stimulates OTRs establishing a positive feedback loop resulting in hypertrophy of the oxytocinergic neurons, decreased astrocytic coverage of neurons, and a subsequent increase in juxtaposition of neurons at the level of somas and dendrites across the entire OT system (for a review see [30]). Ultimately, this process results in a lasting increase in the productivity and functionality of the OT system, the duration of which can last for months depending on the magnitude and length of the stimulation [31]–[33]. Importantly from a therapeutic perspective, these changes can be induced both *in vivo* and *in vitro* by administration of exogenous OT [34]–[37]. Of particular relevance to the present study, plasticity has been demonstrated in the hypothalamic magnocellular neurons of adolescent male rats [38] and robust changes in OTR density occurs in a number of brain regions during adolescent development [39].

Adolescence is a key developmental epoch in mammals during which sexual maturity is attained and adult behavioral repertoires rehearsed and consolidated. Many human psychiatric problems have their ontogeny in perturbations in adolescent development caused by trauma or drug and alcohol abuse [40], [41]. Accordingly, the present study sought to characterize the potential of OT administration, when given chronically during adolescence, to modulate social behavior, anxiety, and alcohol consumption. Alcohol was of interest given the ample evidence that both centrally and peripherally administered OT can reduce tolerance, dependence and self-administration of various drugs of abuse through interaction with neural sites implicated in the development of drug addiction and craving [18], [42]–[46]. Several studies report lower tolerance to and consumption of alcohol amongst breastfeeding mothers (in whom the central OT system is upregulated) compared to non-lactating mothers [47]–[49]. OT administered both centrally and peripherally during chronic ethanol treatment attenuates the development of tolerance to ethanol-induced hypothermic, myorelaxant and akinesic effects in mice [50], [51], and decreases the severity of withdrawal symptoms in mice [52]. In rats, peripheral OT attenuated tolerance to alcohol-induced narcosis [53].

We therefore predicted that rats given repeated OT treatment during adolescence might exhibit altered alcohol self-administration in adulthood. To test this hypothesis we utilized the beer model, a well-established and ecologically valid model of alcohol consumption [54]–[57], which has been successfully used to test a number of pharmacotherapies for alcohol consumption and alcohol-related disorders [58]–[60]. Given that OT inhibits the development of ethanol tolerance (see above), and inhibits drug-induced activation of addiction-relevant brain regions [43], we predicted that OT pre-exposure might affect alcohol consumption. However, we hypothesized that differences in consumption might only emerge towards the end of a long period of continuous alcohol access as it can take several weeks for tolerance [61], [62] and ethanol-induced neuroadaptations to occur [63] with voluntary consumption. We additionally predicted that OT pre-treatment might alter anxiety and social behavior, and be accompanied by long-term up-regulation of endogenous OT systems.

## Methods

### Subjects

All experimental procedures were conducted in accordance with the Australian Code of Practice for the Care and Use of Animals for Scientific Purposes (7^th^ Edition, 2004) and were approved by University of Sydney Animal Ethics Committee (approval number L29/11-2009/3/5178). The subjects were 48 male Australian Albino Wistar (AAW) rats (Animal Resources Centre, Perth, WA, Australia). They were brought to our facility at PND 21 and handled extensively for 11 days prior to treatment. All rats were housed 8 per cage with *ad libitum* access to food and water. The rats were at PND 33 at the start of dosing, an age corresponding to early adolescence. At this time they weighed 127–177 g.

### Drugs

OT was obtained from Auspep Ltd (Parkville, Victoria, Australia) and was dissolved in 0.9% saline vehicle at a concentration of 1 mg/ml and injected intraperitoneally (IP) at a dose of 1 mg/kg. This dose was chosen as several studies have demonstrated long lasting changes following repeated peripheral administration of this dose of OT [26]–[29].

### Experimental design

**Figure 1:**
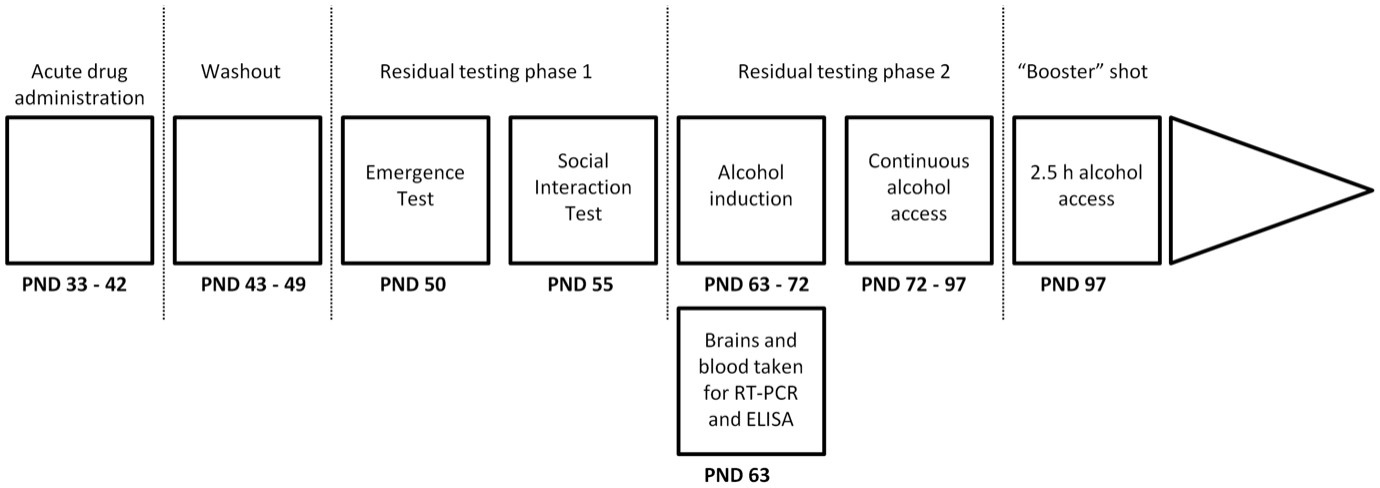
Experimental procedures. There were 24 VEH and 24 OT subjects for inital drug administration, washout and residual testing. Eight VEH and Eight OT subjects from the initial 48 subjects then underwent alcohol induction on PND 63–72 and were used in the subsequent experiments conducted between PND 72–97. Another five VEH and five OT subjects taken from the initial 48 subjects were culled on PND 63 and their brain and blood was later analysed. Abbreviations: VEH = vehicle; OT = oxytocin; PND = post natal day.

An overview of the experimental protocol is presented in Figure 1. Drug administration was conducted between PND 33–42 as this is widely considered to correspond to the early adolescent period in the developmental life cycle of the rat [64], [65]. This period was of particular interest as adolescence is considered a key developmental epoch in humans, during which many psychological and addictive disorders have their roots [40], [41].

The eight day washout before the start of testing was implemented to allow the acute effects of the drug to completely dissipate and to allow the subjects to reach late adolescence. Late adolescence was of particular interest as it is the developmental period during which many psychological disorders, such as generalized and social anxiety become manifest in humans [40]. Furthermore, this washout period allowed the average body weight of the two conditions to return to the same level after transient inhibition of weight gain in the OT-treated rats. The social interaction test was conducted five to six days after the emergence test to minimize possible carry forward effects from one test to the other (see Figure 1).

The brain and plasma analysis was conducted on samples taken on PND 63 as this corresponds to early adulthood in the rat [64], [65] and it was of particular interest to see if OT pre-treatment was capable of causing physiological and neural changes that would endure into adulthood. Alcohol induction also began at PND 63 as, in humans, young adulthood is the period during which excessive patterns of alcohol consumption usually develop [66]. Furthermore, previous research has identified this as a key phase for the development of excessive alcohol consumption in rats [65].

### Drug administration phase

Rats were treated daily with OT 1 mg/kg (group referred to as OT, n = 24) or vehicle (group referred to as VEH, n = 24) for 10 days, from PND 33–42. Body weight was recorded four times during the drug administration phase to establish any effect of OT on weight gain. Subjects were also weighed before the start of the critical tests conducted in the post-drug administration phase to ensure that average weight did not differ significantly between conditions. Body weight was measured throughout the alcohol administration period to ensure there were no differences in body weight which might explain any differential consumption of alcohol.

### Post –drug behavioral testing: Emergence test

After an 8 day washout period, all rats were tested in the emergence test. This was conducted as previously described [67] in an arena measuring 200 (W)×200 (L)×80 (H) cm made of white wooden walls and a black floor. A red Perspex hide box measuring 23 (W)×22.5 (L)×14.5 (H) cm, with a single opening at its center, was placed flush against the center of one of the walls. Testing took place under two bright white spotlights shining directly onto the arena from opposite sides. A camera mounted to the ceiling directly above the apparatus fed video of the tests to a computer and monitor in an adjacent room where the sessions were scored electronically by automatic tracking software (TRACKMATE 1.0; MotMen Ltd, Cook Hill, NSW, Australia). The tracking accuracy of the software has been validated over several years of use in our laboratory with the version used for this study validated using a large cohort of rats (n = 64) and two experienced hand scorers.

The test commenced with individual rats being placed alone inside the hide box by the experimenter. Over a 5 min period the software automatically scored the distance travelled by each subject during the session as well as the time spent (a) in the hide box, (b) with head protruding outside the hide box, (c) in the open field, and (d) in the third of the arena farthest from the hide box (far zone). Additionally, the number of rats to emerge from the hide box in each condition was compared across the two groups. At the end of the 5 min the rat was returned to its home cage and the hide box and arena were thoroughly cleaned with 70% ethanol and allowed to dry before the next rat was tested.

### Post –drug behavioral testing: social interaction test

Five to six days after the emergence test, and 13 days after the end of drug treatment, rats were tested in the social interaction test. The test arena measured 200 (W)×200 (L)×80 (H) cm and consisted of black walls and a black floor. Testing took place under red light. A camera mounted to the ceiling directly above the apparatus projected video of the tests to an external computer and monitor where the sessions were scored electronically by automatic social tracking software (TRACKMATE SOCIAL v. 0.9; MotMen Ltd, Cook Hill, NSW, Australia). The social tracking capability of this software has been validated using a large cohort of rats (n = 64) and two experienced hand scorers.

Social interaction testing was conducted on a single day when the rats were at PND 55. Each rat was matched with a rat of the same experimental condition (OT or VEH) and body weight (within 10 g) but from a different home cage, making a total of 12 pairs from each condition. Each pair was placed in the centre of the arena for 5 min and the amount of social interaction and distance between the two rats over time was automatically scored by the software. The dependent variables of interest were the time animals spent in close proximity to each other (within 1.5 body lengths), the number of active social contacts over the session (these include instances of following, head to head investigation, anogenital investigation and adjacent lying), the average distance travelled for each pair (travelled activity), and the average body movement while stationary for each pair (non-travelled activity). The arena was thoroughly cleaned with 70% ethanol and allowed to dry before the next pair was tested.

### Post –drug behavioral testing: alcohol consumption

A total of 21 days after cessation of OT or VEH pre-treatment (PND 63), 16 rats, 8 per group, were randomly selected to test adult consumption of beer. Rats were initially introduced to alcohol using a lickometer system (as described in [57]) over 9 days of testing. Briefly, this apparatus contains 16 individual chambers that each contained two tubes through which solutions are delivered. The rat licked the tube a certain number of times to receive a predetermined quantity of the solution. In this case, a 0.07 ml drop of solution was provided after every 3 licks at a tube.

A step up procedure was used where the concentration of alcohol in the beer was gradually increased over days. The base solution used was Coopers Ultra-Light 0.44% Alcohol Beer (“near-beer”). Ethanol was then added to the “near-beer” to make 1.44%, 2.44%, 3.44%, and 4.44% ethanol containing beer. “Near-beer” was used as an initial low-alcohol control for any non-specific treatment effects on appetite or taste preference [58]. We have shown that even high levels of “near-beer” consumption do not produce behavioral changes indicative of intoxication [56]. Both tubes in each of the 16 lickometer cages were filled with: “near-beer” on day 1, 1.44% beer solution on day 2, 2.44% beer solution on day 3, 3.44% beer solution on day 4, 4.44% beer solution on days 5–9. Daily sessions in the lickometer ran for 70 min.

Following the ninth and final lickometer session, on PND 72, rats were placed in individual housing where they had 24 h access to 4.44% beer solution, and *ad libitum* food and water for 25 days. Water and beer bottles were weighed and changed at the same time each day (in the middle of the dark cycle) and mls consumed were calculated.

Following the final measurement of intakes on day 25 (PND 97), rats in the OT group received a further 1 mg/kg “booster” injection of OT, while control rats received an equivalent saline injection. Ten minutes after injection rats were placed back in their home cage with access to 4.44% beer solution and water. Two-and-a-half hours later the mls consumed of water and beer were calculated so that the effects of the “booster” shot of OT on beer intake could be assessed.

### Blood collection and brain dissection

At PND 63, 21 days after the end of drug treatment, 10 rats (5 OT & 5 VEH), that had not been exposed to alcohol, were decapitated using a guillotine. Immediately after decapitation, trunk blood for each rat was collected and each brain was dissected, and the hypothalamus was removed, snap-frozen in liquid nitrogen then stored at −80°C as previously described [68]. Trunk blood for each rat was placed into Lithium Heparin tubes and stored on ice before all samples were centrifuged at 1000× g for 10 min at 4°C. Blood plasma supernatant for each sample was collected from the spun tubes and stored individually at −80°C as previously described [69].

### RT-PCR

Each of the 10 frozen hypothalamic tissue samples was homogenized using a rotor-stator homogenizer and RNA was extracted using the RNeasy Mini Kit (QIAGEN, Doncaster, VIC, Australia) according to the manufacturer's instructions. The quality and concentration of extracted RNA was determined using a Nanodrop 2000 spectrophotometer (ThermoScientific, Scoresby, VIC, Australia). Samples were stored at −20°C. RNA was reverse transcribed to cDNA for each sample according to protocols from Applied Biosystems (Mulgrave, VIC, Australia) in a 20 µl reaction containing: 2 µl of 5× buffer RT; 0.33 µl of DTT; 0.33 µl RNasin; 1 µl of 10 mM dNTP mix; 1 µl of 1 mg/ml random primers; 0.5–1 µg of template RNA (quantity dependent on concentration of RNA); and RNase free water (quantity dependent on concentration of RNA). Samples also contained 1 µl of reverse transcriptase whereas negative controls contained none. The reaction mix was incubated in a gradient thermal cycler at 37°C for 1 h then at 70°C for 15 min to inactivate reverse transcriptase. Samples were stored at −20°C.

cDNA was prepared for amplification according to protocols from Applied Biosystems (Mulgrave, VIC, Australia) on a 96 well reaction plate. Each 25 µl reaction contained: 12.5 µl of Taqman Universal PCR Master Mix, No AmpErase UNG (2×); 1.25 µl of 20× 18S Taqman Endogenous Control Mix; 1 µl of cDNA; 9 µl of water. Finally, each 25 µl reaction contained 1.25 µl of the relevant 20× Taqman Gene Expression Assay Mix: rat OT gene expression assay or rat OTR gene expression assay.

Following preparation, the plate was centrifuged at 10,000 rpm for 30 s at 25°C. Relative quantification was then performed using real time PCR on ABI PRISM 7000 detection system (Applied Biosystems, Mulgrave, VIC, Australia) using thermal cycling conditions consisting of an initial denaturation stage of 10 min at 95°C, then 40 cycles at 95°C for 15 s, annealing at 60°C for 1 min. Two negative controls were included, one containing no cDNA template, the other containing the negative control mix from the reverse transcription stage. Reactions for each biological sample and the negative controls were run in triplicates. Two plates were run in total, one for each target gene of interest: OT and OTR.

### ELISA

Determination of plasma OT concentration was performed as using an Oxytocin Enzyme Immunoassay Kit (Assay Designs, Ann Arbor, MI, USA) according to manufacturer instructions. The only additional substance required was a protease inhibitor (Sigma-Aldrich, Castle Hill, Australia), which was added to the samples, and deionised water. All samples were run in duplicate and standards were prepared to allow determination of concentration. Prepared plates were analyzed using a microplate reader and analysis involved comparisons of the unknown samples with a standard curve generated from internal standards. Results were expressed as the plasma concentration of OT in pg/ml for each sample.

### Statistics

Body weight data were analysed using mixed model ANOVA with trend analysis of the dosing period used to establish differences in weight gain and follow up contrasts in the post-treatment period used to verify that the two groups returned to and remained at the same weight after the cessation of treatment.

Data from the emergence test and social interaction tests were analysed using independent samples t-tests. Additionally, the number of rats to emerge from the hide box in each condition was compared using a Chi-Square Test of independence. Pattern of beer and water consumption over the 25 days home cage access were examined using mixed model ANOVA and planned contrasts were conducted to compare the intake between the two groups on each day. Given the number of comparisons required, a decision wise error rate (DER) was used as is recommended by Perneger [70]. Total beer and water consumed over the 25 days, the last week, and in the 2.5 h following the “booster” shot were examined using independent samples t-tests.

The analysis for the RT-PCR was conducted using ABI PRISM 7000 detection system software 7000 SDS version 1.3.1.21 (Applied Biosystems) to obtain CT values for gene expression and statistical analysis was conducted using Relative Expression Software Tool (REST) version 2.0.7 (Corbett Research Pty. Ltd.). Relative gene expression was analysed for statistical significance for each normalised target gene (OT and OTR) comparing the OT pre-treated samples to the VEH samples.

For the ELISA the average OT plasma concentration was compared across groups using an independent sample t-test.

Welch's correction for unequal variances was used wherever the assumption of homogeneity of variance was violated as indicated by a p-value<0.05 on Levene's test for homogeneity of variance.

## Results

### Body weight

At the start of dosing there was no difference in body weight between OT (*M = *150.37, *SD = *8.90) and VEH (*M = *150.83, *SD = *11.29) rats, *p* = 0.877. However over the dosing period weight increased at a significantly greater rate for VEH compared to OT rats, as indicated by the significant linear interaction trend, F(1,46) = 7.34, p<0.01. However, despite this difference in weight gain there was no significant difference in mean weight between OT (*M = *203.77, *SD = *13.01) and VEH (*M = *211.08, *SD = *19.84) rats on the last day of treatment, *p = *.138. There was no significant difference in body weight between OT (*M = *297.79, *SD = *15.73) and VEH (*M = *301.71, *SD = *21.17) rats at the start of behavioral testing, 8 days after the cessation of dosing, *p* = 0.471. Furthermore, the groups did not differ significantly at any timepoint throughout the alcohol administration period (all *p-values>*0.05), with OT (*M = *442.50, *SD = *30.90) and VEH (*M = *450.62, *SD = *20.37) subjects equivalent in weight at the end of the experiment when the final “booster” shot was given *p = *0.546.

### Emergence test

**Figure 2:**
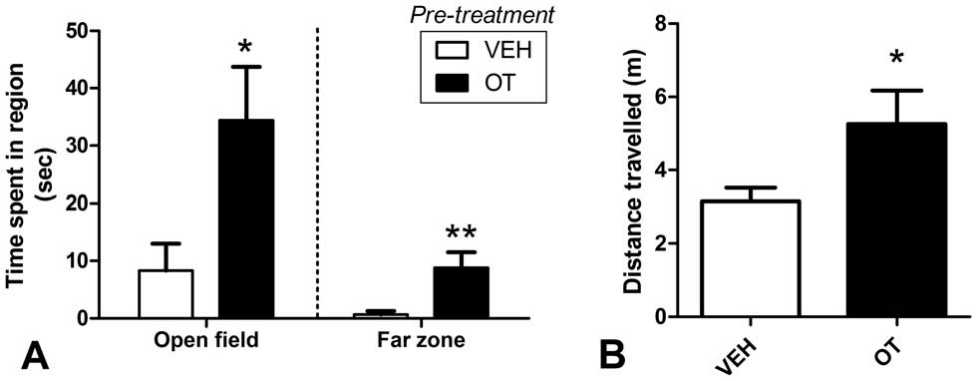
Emergence test. Results from the 5 min emergence test conducted 8 days after the cessation of 10 consecutive days of once per day treatments with either 1 mg/kg OT or VEH. **A.** Mean (+SEM) seconds spent in the open field and in the third of the open field farthest from the hide box (far zone). **B.** Mean (+SEM) metres travelled during the test. N = 24 per condition. * Significantly different to VEH, p<0.05 ** Significantly different to VEH, p<0.01.

The Chi-Square test of independence revealed significantly more OT pre-treated rats emerged from the hide box during the 5 min session (15 out of 24) compared to VEH treated rats (4 out of 24), χ^2^(48) = 10.54, p = 0.001. Rats pre-treated with OT spent significantly: less time in the hide box [*t*(35.827) = 2.30, *p* = 0.027]; more time in the open field (Figure 2A) [*t*(34.256) = 2.51, *p* = 0.017]; and more time in the far zone of the open field (Figure 2A), *t*(24.995) = 2.96 *p*<0.01. Furthermore, the OT pre-treated rats travelled significantly more distance during the session compared to the VEH pre-treated animals (Figure 2B), *t*(28.89) = 2.12, *p* = 0.043. There was no significant difference between OT and VEH animals in time spent with head out of the hide box, p>0.05.

### Social interaction test

**Figure 3:**
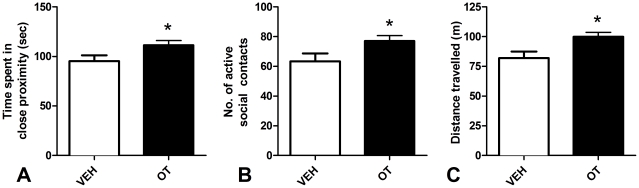
Social interaction test. Results from a 5 min social interaction test conducted 13 days after the cessation of 10 consecutive days of once per day treatments with either 1 mg/kg OT or VEH. **A.** Mean (+SEM) seconds the two subjects spent in close proximity (within one body length). **B.** Mean (+SEM) number of active social contacts between the subjects. **C.** Mean (+SEM) meters travelled by each pair of subjects (on average) during the social interaction test. N = 24 per condition. * Significantly different to VEH, p<0.05.

OT pairs spent significantly more time than VEH pairs in close proximity to each other (Figure 3A), *t*(22) = 2.17, *p* = 0.041. OT pairs also came into social contact significantly more times than VEH pairs (Figure 3B), *t*(22) = 2.12, *p* = 0.045. As seen in Figure 3C, OT rats travelled significantly more meters than VEH animals during the social interaction test, *t*(22) = 2.68, *p* = 0.014.

### Consumption during alcohol induction and continuous access to beer

**Figure 4:**
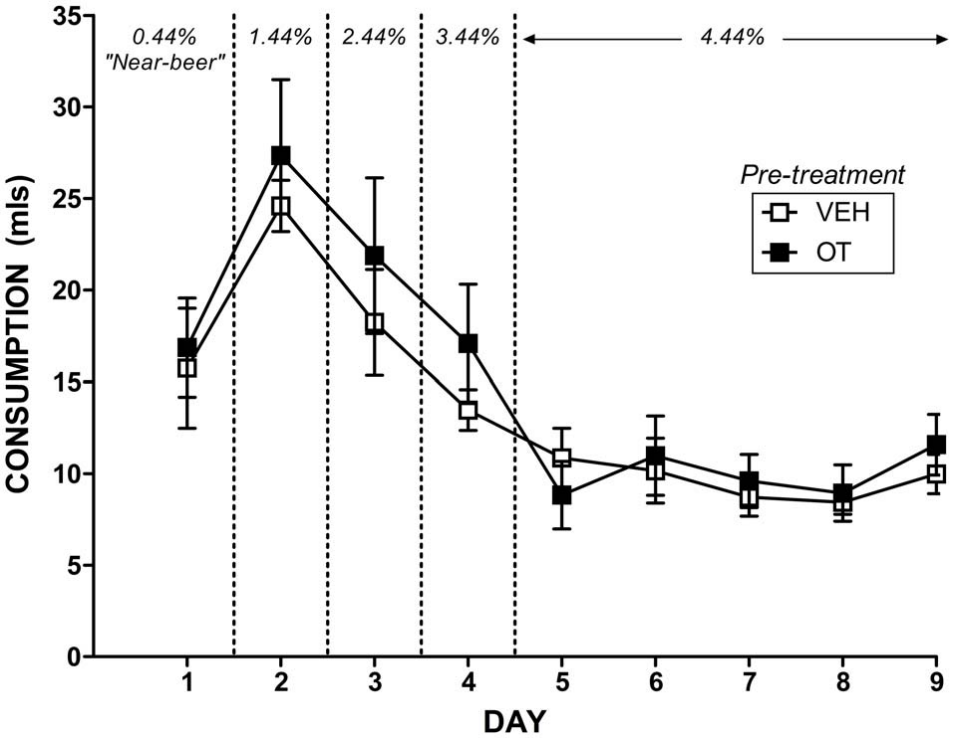
“Near-beer” and beer consumption during the 9 day alcohol induction phase in the lickometer apparatus. Mean (± SEM) consumption (in mls) of increasing concentrations of beer (from .44% “near-beer” up to 4.44% EtOH beer). The 9 day induction period began 21 days after the cessation of 10 consecutive days of once per day treatments with either 1 mg/kg OT or equivalent VEH saline. Subjects were placed in the lickometer for one 70 min session per day. N = 8 per condition.

During the alcohol induction period in the lickometer apparatus, during which alcohol concentrations were gradually increased in daily 70 min sessions, there were no significant differences between OT and VEH pre-treated subjects in the consumption of any ethanol concentration of beer (see Figure 4): “near-beer” (.44%) (*p* = 0.794); 1.44% (*p* = 0.556); 2.44% (*p* = 0.490); 3.44% (*p* = 0.320); or 4.44% (all *p-values*>0.421).

**Figure 5:**
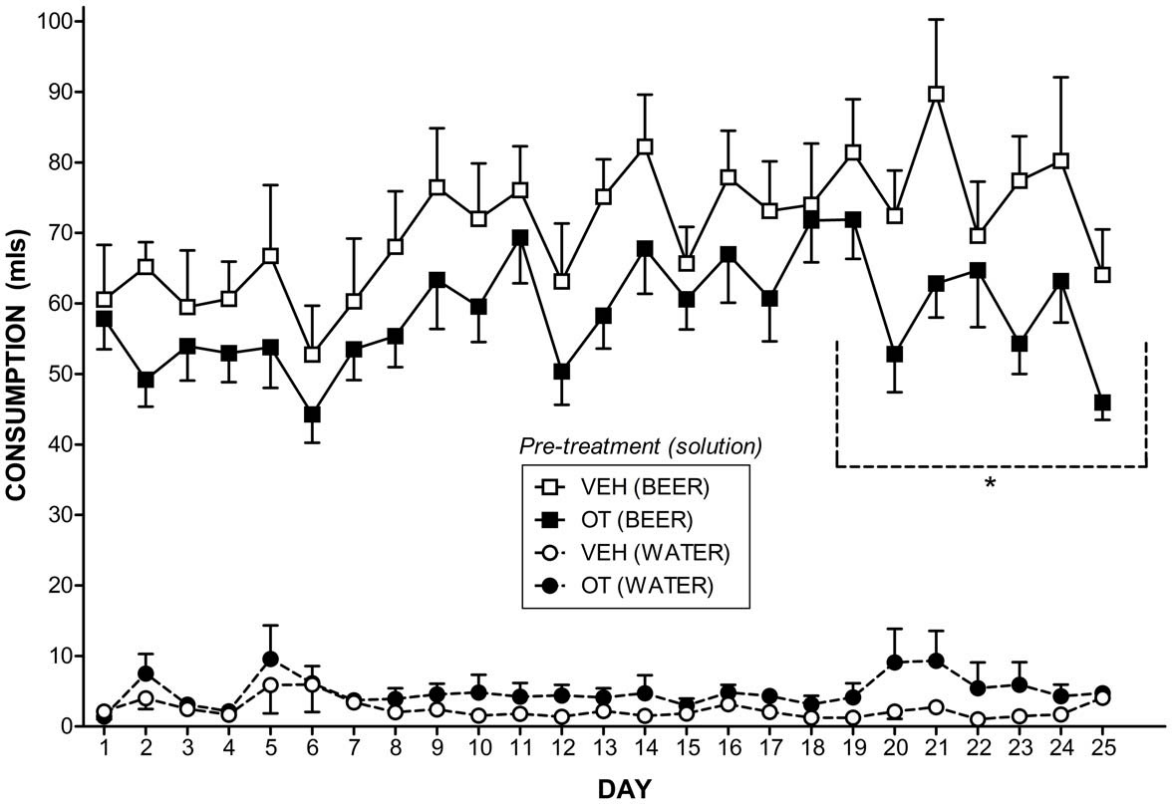
Alcohol and water consumption under continuous access. Mean (± SEM) 4.44% EtOH beer and water consumption (in mls) for each of the 25 days of continuous access to beer and water is illustrated. Continuous access began 30 days after the cessation of 10 consecutive days of once per day treatments with either 1 mg/kg OT or equivalent VEH. Subjects were housed individually and had 24 h access to both beer and water over the entire 25 days. N = 8 per condition. * indicates sig. difference in total beer consumption between OT and VEH over the final seven days of continuous access, p<0.05.

Figure 5 illustrates the consumption for OT and VEH pre-treated subjects over the course of the continuous access period for beer and water under individual housing. There was no significant overall pre-treatment effect on beer or water consumption, both *p-values*>0.05. However, the OT pre-treated animals consumed significantly less beer than the VEH pre-treated animals on day 2 [*F*(1, 14) = 9.55, *p*<0.01], 13 [*F*(1, 14) = 5.71, *p* = 0.031], 20 [*F*(1, 14) = 5.47, *p* = 0.035], 21 [*F*(1, 14) = 5.34, *p* = 0.037], 23 [*F*(1, 14) = 9.08, *p*<0.01] and 25 [*F*(1, 14) = 6.84, *p* = 0.020] of continuous access.

Furthermore, over the final seven days of continuous access OT pre-treated rats consumed significantly less beer in total than the VEH pre-treated rats, *t*(14) = 2.24, *p* = 0.042. This emerging difference was further reflected by VEH animals alcohol intake showing a significant linear increase over the 25 days [*F*(1, 7) = 7.85, *p* = 0.026] and OT animals showing no such increase, *F*(1, 7) = 3.47, *p* = 0.105. OT pre-treated animals consumed significantly more water than VEH pre-treated animals on day 17 [*F*(1, 14) = 7.12, *p* = 0.018] but there were no significant differences in water consumption on any other days, all *p-values*>0.05.

### Effects of an OT “booster” shot on beer consumption

**Figure 6:**
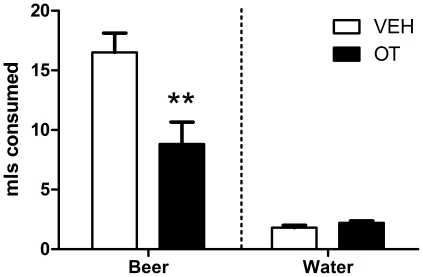
Alcohol and water consumption following the OT “booster” shot. Mean (+SEM) consumption (mls) of 4.44% EtOH beer and water over the 2.5 h following injection of a “booster” of either 1 mg/kg OT or equivalent VEH saline. The “booster” shot was administered after the final measurement of intakes on the final day of continuous access to beer and water. N = 8 per condition. ** Significantly different to VEH, p<0.01.

Figure 6 illustrates consumption of beer and water in the 2.5 h following a “booster” treatment with OT or VEH saline. Following the “booster” dose OT subjects consumed significantly less beer than VEH subjects, *t*(14) = 3.13, *p*<0.01. There was no significant difference in water consumption between OT and VEH subjects, *p*>0.05.

### RT-PCR

**Figure 7:**
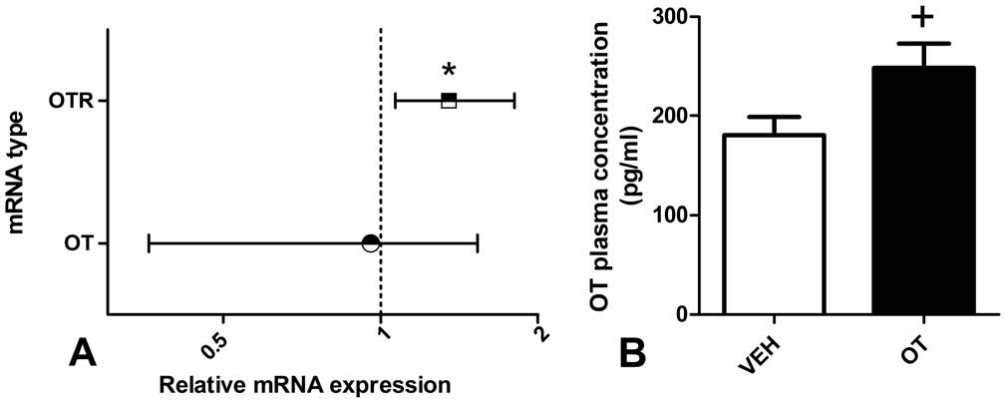
RT-PCR and ELISA. Results from the RT-PCR and ELISA conducted on the 10 rats (5 OT, 5 VEH) randomly selected 21 days after the cessation of 10 consecutive days of once per day treatments with either 1 mg/kg OT or equivalent VEH saline. **A.** Fold change (±SE) in OT and OTR mRNA expression in the OT pre-treated subjects compared to VEH. **B.** Mean (+SEM) blood plasma concentration of OT (pg/ml) in the OT and VEH pre-treated subjects. *Significant fold change, p<0.05. + Trend towards significant difference compared to VEH, p = 0.057.

All samples were clean and had adequate RNA concentration (data not shown). There was no amplification in negative controls, indicating expression in biological samples was not due to contamination. OTR mRNA was significantly up-regulated in OT animals to 1.35 fold higher expression than the VEH animals (Figure 7A), *p* = 0.032. OT mRNA expression was essentially equal in OT and VEH animals (fold difference = 0.958), *p*>0.05.

### ELISA

In the cohort of adolescent Wistar rats, there was a strong trend towards significantly greater plasma OT concentration (pg/ml) (Figure 7B) in the OT compared to VEH animals, *t*(8) = 2.22, *p* = 0.057.

## Discussion

The current results provide intriguing preliminary evidence that repeated exposure to exogenous OT in adolescence can have subtle yet significant effects that last into adulthood, effects that are potentially significant in a clinical sense. These effects include (1) reduced anxiety, (2) increased sociability, (3) decreased alcohol consumption, and (4) up-regulated OT in plasma and OTR mRNA in the hypothalamus. In addition, we demonstrated that a “booster” shot of OT can have an additional acute inhibitory effect on alcohol consumption. These effects are now discussed in turn.

### Inhibited body weight gain over dosing period

Over the 10 day dosing period OT treated rats gained weight at a significantly slower rate compared to VEH treated rats. Previously reported effects of OT on body weight gain are somewhat contradictory with studies reporting both increases and reductions in weight gain after OT administration [71], [72]. Reductions in weight gain or weight loss have been associated with an acute decrease in food intake for several hours following OT administration [72]. Increases in weight gain following OT administration are not associated with any change in food intake but rather with neuroendocrine changes that promote anabolic metabolism [71]. Most likely there are strain and possibly sex and developmental differences that dictate the effect of OT on weight gain which may depend on factors such as endocrine profile and sensitivity to OT. Weight quickly recovered and at the start of behavioral testing, one week after the cessation of OT administration, there was no weight difference between OT and VEH subjects and this lack of difference remained throughout the study. This suggests the effect of OT on weight gain is transient and caused by the direct effects of the exogenous OT [73].

### Reduced generalized anxiety

More than one week following the cessation of pre-treatment the subjects given OT showed a marked reduction in generalized anxiety-like behavior in the emergence test. Additionally, over 2 weeks after cessation of pre-treatment, subjects given OT had increased sociability and activity in the social interaction test, with increases in locomotor activity in open-fields indicative of increased exploratory behavior and reduced general-anxiety [74], [75]. OT pre-treated subjects also travelled significantly more distance in the emergence test which is to be expected given more of the OT pre-treated subjects emerged from the confines of the hidebox. However, given that an increase in locomotor activity was also observed in the social interaction test, an effect of OT pre-treatment on general locomotor activity cannot be ruled out.

The reductions in generalized anxiety-like behaviour observed in this study are in line with a variety of studies demonstrating anxiolytic properties of OT and OT receptor agonists [7], [9], [11], [21]. However, perhaps of most interest, the present findings confirm that OT can cause sustained reduction in general anxiety-like behavior that remains well beyond the period during which the administered OT is present. Several studies have found an anxiolytic effect of OT only after chronic administration [24], [25] and in light of the present study's findings it is plausible that the anxiolytic effect of OT is due as much to lasting behavioral and neuroadaptations as it is to the direct effects of administered OT, which are presumably present for only a short time.

This longer-term reduction in anxiety caused by the short term treatment with OT is consistent with previous reports of altered physiological functioning (such as decreased blood pressure and corticosterone blood concentration) that lasted well beyond the duration of the treatment [10], [26]–[29], [71], [76]. Interestingly, all of those physiological changes are associated with a relaxed condition [77]–[82]. This suggests OT may be having both a psychological and physiological impact that is conducive to a lasting state of reduced anxiety.

### Increased sociability

More than two weeks after the cessation of pre-treatment the subjects given OT showed a subtle but significant increase in social behavior indicated by a greater frequency of active social contacts and an increased duration spent in close proximity in the social interaction test compared to VEH treated subjects. Consistent with the assertion that OT plays an important role in the formation and regulation of social bonds [83], [84], these findings support previous research that indicated exogenous OT causes a reduction in social anxiety and lessens the impact of social isolation by perhaps mimicking social interaction itself [12]–[15]. Furthermore, the present study extends our understanding of the effects of exogenous OT on social behavior by providing initial evidence that peripherally administered OT can cause longer term adaptive behavioral changes that are conducive to reduced social anxiety and a subsequent increase in initiations of positive social contact.

### Alcohol consumption

During the 9 day alcohol induction period there were no significant differences between OT and VEH pre-treated rats in consumption of varying concentrations of beer. Importantly, the lack of a significant difference in the consumption of the “near-beer” base solution establishes that the later diminution in the consumption of alcoholic beer as a result of OT pre-treatment is unlikely to be due to basic alterations in appetite or taste aversion as the “near-beer” differs from the higher concentration beer only in EtOH content [58]. Furthermore, the lack of a significant difference in weight between the groups throughout the alcohol induction and continuous access stage of testing provides further evidence that there was no difference in caloric intake between the two conditions.

During the 25 days of continuous access to beer and water in the home cage, which started 30 days after the cessation of drug administration, a significant difference emerged over the access period with OT pre-treated rats consuming significantly less alcohol over the final week of continuous access. In line with this, the alcohol consumption of VEH rats increased significantly over the course of the experiment, whereas there was no significant increase for OT rats. This provides preliminary evidence that short-term pre-treatment with OT can inhibit the development of alcohol consumption for at least 55 days after the cessation of the pre-treatment regime. This is particularly exciting given the short action of currently available treatments for alcohol abuse leads to a very high rate of relapse resulting in a great need for treatment options with improved long term efficacy, such as that demonstrated by OT in this study [54], [85], [86].

Interestingly, as we predicted, the emergence of a stronger, more convincing, difference in alcohol intake between VEH and OT rats from day 19 to 25 coincides with the time-course for development of tolerance to alcohol in standard strain and non-alcohol preferring rats (sometimes taking up to 24 days or longer) [61], [62]. Previous studies have demonstrated OT and OT fragments are able to reduce or eliminate tolerance to a number of the behavioural and physiological effects of ethanol [50], [51], [53], [87], [88]. This suggests one possibility is that the emerging consummatory differences between VEH and OT rats in the present study may be due to absence of tolerance in the OT-treated subjects. Studies from the human literature also provide anecdotal support for this theory with several studies reporting lower tolerance to and consumption of alcohol amongst breastfeeding mothers, in whom the central OT system is up-regulated compared to non-lactating mothers [47]–[49]. Alternatively, the emerging difference in ethanol consumption observed in this study may be due to inhibition of addiction related neuroadaptations causes by ethanol in regions such as the Nucleus Accumbens. This is plausible given that it can take several weeks for ethanol induced neuroadaptaions in brain regions involved in addiction to occur [63] and OT has been shown to inhibit drug induced activation of these regions [43]. Future studies should explore the possibility that OT induced differences in ethanol consumption are due to inhibition of the development of tolerance and/or changes in the actions of ethanol in brain regions involved in addiction.

Of importance, this study established that OT can cause long-term reductions in alcohol consumption using a continuous access paradigm. Demonstrating such effects under continuous access is particularly challenging due to the constant availability of alcohol. However, it is also arguably the most valid model given the widespread current availability of alcohol in human society. Furthermore, the access was provided in individual housing where there was no other source of stimulation for the rat. Such an environment is known to be stressful, and given the direct relationship between stress and alcohol consumption (for a review of the negative impact of isolation on stress levels and alcohol consumption in rats see [89]) this provided further ecological validity to the environment in which alcohol was provided. For example, it has been shown that socially isolated rats sometimes consume a significantly greater quantity of ethanol than group housed rats [90]. It is therefore possible that OT pre-treatment is augmenting alcohol intake by preventing stress induced increases in alcohol intake arising from the isolation housing. The fact that OT effects were found under a paradigm so conducive to alcohol consumption and so long after treatment is quite remarkable.

### OT “booster” shot

In addition to the long-term suppression of alcohol consumption seen after chronic OT administration, the “booster” shot of OT at the end of the 25 day continuous access period (when consumption patterns were established) significantly inhibited consumption of alcohol for the 2.5 h following treatment. Furthermore, the “booster” treatment had no effect on the consumption of water over the 2.5 h period, which suggests it might be an effect that is selective to alcohol containing fluids. This provides evidence that acute OT could be an effective and selective treatment aimed at immediate reductions in alcohol consumption as well as an effective option for more long-term term reductions in consumption. However, the present study does not determine whether the pre-treatment is required for the more immediate effects of the “booster” shot to occur. The current study therefore provides impetus for more comprehensive future preclinical studies of OT as a possible therapeutic for alcohol use disorders. Future studies might utilize “near-beer” as a control solution (as described previously in [58]) to determine with greater precision how specific the consummatory suppression resulting from the “booster shot” is to alcohol.

### Enduring upregulation of the endogenous OT system

As expected, there was evidence of long-term enhanced functioning of the central OT system in response to peripheral OT treatment. OTR mRNA was significantly, albeit moderately, upregulated and there was evidence of increased plasma levels of OT. This is not surprising given the essential role OTRs play in inducing and maintaining long-term neuroadaptations of the OT system [30]. For example, increased OT levels (both central and peripheral) and increased OTR binding, mRNA and density has been associated with the up-regulation of the OT system that occurs during gestation, parturition and lactation [30], [91]–[94]. It is plausible that upregulation of OTR mRNA is at least partly responsible for the behavioral findings in this study given the association between increased OTR mRNA and reduced anxiety [95], and increased OT levels and OTR binding and reduced alcohol consumption [96], [97].

There was a strong trend towards increased OT plasma concentration in OT pre-treated rats in this study and given blood collection took place 21 days after treatment, it is possible the effect was simply wearing off and would have been significant closer to treatment. Follow up analysis of the plasma from another study in which rats underwent a similar pre-treatment with OT or VEH found a highly significant increase in OT plasma levels in the OT pre-treated animals two weeks after the cessation of the pre-treatment period (Carson, Bowen & McGregor, unpublished findings). Furthermore, the plasma levels observed in that experiment were similar to those observed here, providing further support for the strong trend observed in the present study. This provides further evidence that chronic OT administration causes a lasting up-regulation of activity in the endogenous oxytocinergic system and is in line with a wealth of previous studies which demonstrate that this system is capable of undergoing morphological changes which create a positive feedback loop whereby increased OT levels stimulate further OT release (for a review see [30]).

### Conclusion

This study has provided initial evidence that OT might have utility as a unique pharmacotherapy with both treatment and prophylactic utility. A brief administration of OT during early adolescence reduced generalized and social anxiety related behaviors in late adolescence and inhibited the development of excessive alcohol consumption in adulthood. These behavioral changes were associated with long-term up-regulation of the endogenous OT system. Finally, a “booster” dose of OT caused an immediate and marked reduction in alcohol consumption. These results suggest OT, or synthetic OT receptor agonists, could have the potential to be a beneficial and enduring treatment option for generalized and social anxiety disorders as well as alcohol use disorders. Current pharmacological treatment options for these disorders are plagued by lack of efficacy and intolerable side effects. The enduring nature of the behavioral and morphological changes seen in this study suggests that exogenous OT may have the capacity to do more than temporarily alleviate pathological symptoms.
